# The biology of bone morphogenetic protein signaling pathway in cerebrovascular system

**DOI:** 10.1186/s41016-021-00254-0

**Published:** 2021-09-01

**Authors:** Haishuang Tang, Xiaoxi Zhang, Gaici Xue, Fengfeng Xu, Qingsong Wang, Pengfei Yang, Bo Hong, Yi Xu, Qinghai Huang, Jianmin Liu, Qiao Zuo

**Affiliations:** 1 Department of Neurosurgery, Changhai Hospital, Naval Military Medical University, 168 Changhai Road, Shanghai, 200433 People’s Republic of China; 2 Naval Medical Center of PLA, Naval Military Medical University, Shanghai, 200050 People’s Republic of China; 3 Department of Cardiology, the First Medical Centre, Chinese PLA General Hospital, Beijing, 100853 People’s Republic of China

**Keywords:** Bone morphogenetic protein, Hemodynamic, Neovascularization, Cerebrovascular diseases

## Abstract

Bone morphogenetic protein belongs to transcription growth factor superfamily β; bone morphogenetic protein signal pathway regulates cell proliferation, differentiation, and apoptosis among different tissues. Cerebrovascular system supplies sufficient oxygen and blood into brain to maintain its normal function. The disorder of cerebrovascular system will result into serious cerebrovascular diseases, which is gradually becoming a major threat to human health in modern society. In recent decades, many studies have revealed the underlying biology and mechanism of bone morphogenetic protein signal pathway played in cerebrovascular system. This review will discuss the relationship between the two aspects, aiming to provide new perspective for non-invasive treatment and basic research of cerebrovascular diseases.

## Introduction

Different from other organs in human, the brain, accounting only for 2% of body weight, needs about 20% circulating blood supplied through cerebrovascular system. Thus, the natural development of vascular embryogenesis is critical in normal function maintenance of cerebrovascular system. The cerebrovascular vessel consists of 3 layers: the intima layer, the median layer, and the outer layer. Under physical condition, the vessel system keeps in a homeostasis state, and the hemodynamic force is in balanced with vessel protective mechanism. The abnormal vascular embryonic development or imbalance between hemodynamic force and vessel protective effect will results into serious cerebrovascular diseases (CVD). In modern society, cerebrovascular diseases, including intracranial aneurysm (IA), cerebrovascular malformation, and ischemic disease, have become one of the main causes of disability and mortality, bringing much economy and psychology burden to families [1, 2].

Bone morphogenetic protein (BMP) is a member of transcription growth factor superfamily β (TGF-β), which was primarily evidenced to promote ectopic bone formation in rats [3]. With the deepening of research, researchers have found that BMP signal pathway plays a respective role in regulating cell proliferation, differentiation, migration, and apoptosis in different diseases and tissues [4]. Previous studies on BMP signaling pathway mainly focused on skeletal system diseases and tumor-related diseases [5, 6]. In recent years, the abnormal expression of BMP signaling pathway has been revealed in a series of vascular diseases [7]. Many reviews have dedicated to describe the relationship between BMP signaling and vascular diseases, and for CVD, less reviews can be retrieved [7, 8]. This review will conclude the recent relevant studies focusing on the relationship between BMP signal pathway and hemodynamics, cerebrovascular embryonic development, and the typical CVD in cerebrovascular system. It is hoped that this review will bring new research perspective for the research and treatment of CVD.

## Overview of BMP signal pathway

### The introduction of BMP

Till now, 33 ligands have been evidenced in TGF-β, and among them, more than 20 ligands belong to BMP superfamily, which can be further divided into BMP-2/4 protein, BMP-5/6/7/8 protein, growth and differentiation factor (GDFs)-5/6/7, BMP-9/10 protein, etc. The activity BMP is regulated and modulated by many molecules both intracellular and extracellular [9]. In physiology state, various BMP signal pathway antagonists exist in different tissues of human body. Normally, these antagonists restrain BMP activity by inhibiting the combination of BMP and its targeted receptor [10]. Under such circumstance, the constant expression of antagonists is conducive to maintain human homeostasis. Once the expression of these antagonists is restrained or in a disorder state, the abnormal BMP signal pathway activation will become one of the initiating factors of various diseases [11, 12]. For cerebrovascular system, BMP-2/3/4/6/7/910 were the most frequent reported by previous researches [13–15].

### BMP receptors and co-receptors on cell membrane

Like other proteins in the TGF-β superfamily, BMP can bind to two kinds of serine threonine kinase receptors, known as BMP receptor I (BMPRI) and BMP receptor II (BMPRII). BMPRI and BMPRII share similar structures, which are composed of intracellular domain, transmembrane domain, and extracellular domain, and the intracellular domain possesses serine threonine kinase activity. The affinity of type I receptor is higher than that of type II receptor, and its receptor affinity is greatly enhanced after the formation of receptor heterotetrameric complexes [16]. In addition to BMPRI and BMPRII, BMP can bind to activin receptor 2A (ACVR2A) and activin receptor 2B (ACVR2B) to activate downstream signaling pathways [17]. ACVR2A and ACVR2B receptors are expressed in many tissues of human body. The signal pathway is then activated when BMP binds to its targeted receptor, initiating the following downstream effect. In addition to BMP receptors, a series of co-receptors have been reported to modulate the activation of BMP signaling. Endoglin and betaglycan are the main 2 co-receptors being evidenced to exert significant role in vascular diseases [18, 19].

### Smad-mediated and non-Smad-mediated BMP signal pathway

**Fig. 1:**
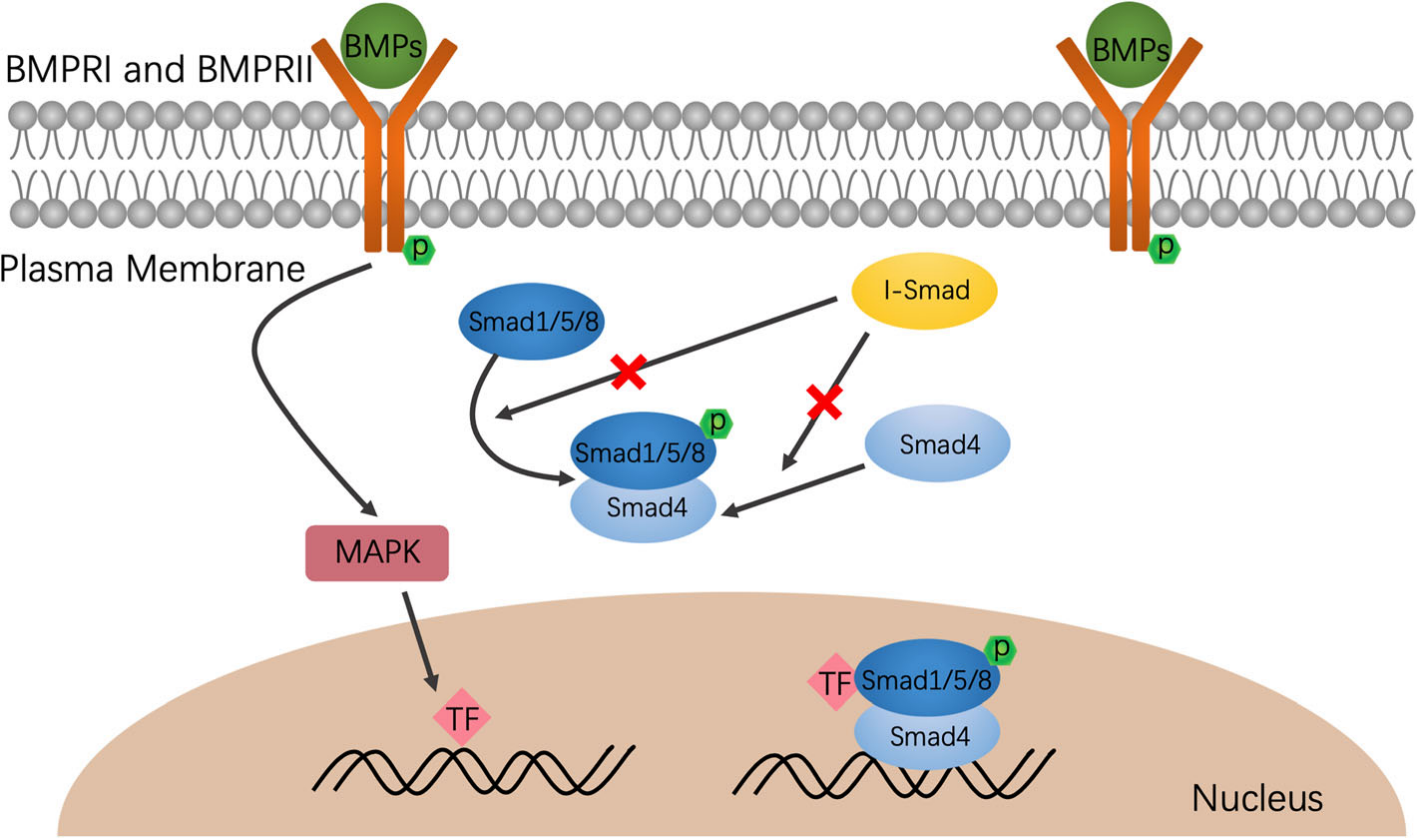
Overview of the BMP signaling pathway. BMPRI and BMPRII are located in the plasma membrane, composed of intracellular domain, transmembrane domain, and extracellular domain, and the intracellular domain possesses serine threonine kinase activity. BMPRI and BMPRII together with BMPs form heterotetrameric complex and translate signal to nucleus. This process can be inhibited by inhibitory Smad. Besides Smad pathway, non-Smad pathway, such as MAPK pathway, can also participate the BMP signal. All the effectors will finally lead to the induction of targeted gene expression

BMP signal pathway mediated via Smad way is the cardinal signal transduction pattern in regulating cell function. According to their specific function, Smad Protein can be divided into receptor regulated Smad (R-Smad), inhibitory Smad (I-Smad), and common mediator Smad [20]. Once the BMP receptor complex is formed, the BMPR II receptor phosphorylates BMPR I receptor. The activated type I receptor activates intracellular signal transduction through R-Smad protein. The R-Smad protein binds with Smad4 protein to form a heteromeric and enters the nucleus, which regulates the transcription response [21]. I-Smad inhibits the activation of downstream signaling pathway of BMP by antagonizing the formation of BMP receptor complex [22]. In addition to the canonical Smad protein signaling pathway, BMP can also mediate downstream signaling through non-Smad way [23]. BMP can transmit signals in different tissues by activating mitogen activated protein kinase (MAPK), protein kinase C (PKC), phosphoinositide 3 kinase (PI3K)/Akt, Erk, etc., which plays an important role in various vascular diseases [24, 25] (Fig. 1).

## BMP signaling pathway and neovascularization

Vascular embryonic development is an important event in the process of embryonic development. The natural process of human vascular embryonic development mainly involves vascular endothelial cells, vascular smooth muscle cells, and perivascular cells, in which the proliferation, migration, and tube wall formation of endothelial cells are crucial during this process. Besides the vascular development from de novo in fetus, angiogenesis, meaning sprouting vessel wall through previous existing vessel, in adults occurs extensively in inflammation, wood healing, and female menstruation [26]. Angiogenesis together with embryonic vascular development are named neovascularization, and BMP signaling pathway are wildly reported to participate in this process [27, 28]. The research shows that BMP-2, BMP-4, BMP-6, and BMP-7 proteins are related to the proliferation and migration of endothelial cells while BMP-9 inhibits the migration of endothelial cells and the angiogenesis induced by vascular endothelial growth factor [14]. Suzuki et al. suggested that in mouse embryos, low dose of BMP-9 promoted the formation of vascular tube formation via activating endothelial proliferation [29]. Richter et al. found that in mice, BMP-9 promotes the sprouting angiogenesis derived from endothelial cells through Smad1/5 activation [30]. Ouarné et al. compared the vessel normalization in BMP-9 knockout mice and BMP-10 knockout mice, and they found that BMP-9 and BMP-10 may play different roles and BMP-9 is comparatively more important in vessel normalization [31]. Thus, the role of BMP-9 played in angiogenesis may depend on different microenvironments and different concentrations.

In addition to endothelial cells, smooth muscle proliferation, differentiation, and migration are also the crucial steps in neovascularization. Researches showed that BMP signaling plays an important role in angiogenesis by regulating smooth muscle cell function [32, 33]. Vascular proliferative disorders are characterized by the proliferation of vascular smooth muscle cells and excessive extracellular matrix synthesis. Nakaoka et al. reported that in rat carotid artery balloon injury model, the transfer of the BMP-2 gene via adenovirus reduced intimal hyperplasia significantly, and they evidenced that BMP-2 could inhibit SMC proliferation and suggested the possibility of therapeutic application of BMP-2 for the prevention of vascular proliferative disorders [34]. Zhang et al. found that in vascular smooth muscle cell, overexpression of BMP-2 via adenoviral way could profoundly augment the smooth muscle cell mobility, and this effect was through the Erk signaling pathway [35]. Other study suggested that BMP-4 could inhibit the proliferation of smooth muscle cells in the proximal pulmonary artery and promoted the proliferation of smooth muscle cells in the distal pulmonary artery [36]. Therefore, similar to endothelial cells, the regulation of BMP signaling pathway on smooth muscle cells also depends on different cell sources and different cell culture environments.

Peripheral cells are embedded in the basement membrane of capillary endothelial cells, communicate with endothelial cells through physical contact and paracrine signals, and monitor and stabilize the maturation process of endothelial cells. The neovascularization process was closely associated with pericytes, especially in the development of blood brain barrier. Lei et al. reported that BMP-3 regulates blood brain barrier integrity in zebrafish brain by promoting pericyte development. And knockdown of BMP-3 was accompanied by intracerebral hemorrhage in zebrafish embryos [15]. Uemura et al. reported that BMP-4, highly expressed in white matter pericytes, promoted angiogenesis, which indicate that of BMP-4 signaling is a potential therapeutic strategy for treating subcortical small vessel disease [37]. These findings evidenced the significance of BMP in regulating the function of pericytes.

## BMP signaling pathway and hemodynamics

Blood flows continuously in the human vascular system to maintain the normal metabolic balance and the stability of the internal environment. The irregular wall shear stress caused by abnormal hemodynamics is often accompanied by a series of vascular disorders. In the intracranial vessels, cerebral aneurysm, arteriovenous malformation, and arteriovenous fistula are closely related to abnormal hemodynamics [38–40]. When the blood flow parameters change, the abnormal wall shear stress is sensed by the cilia and polysaccharide protein complex on the membrane surface of vascular endothelial cells, and the wall shear stress stimulation is transformed into biological signal through the ion channel on the membrane surface. Researchers have shown that the number of cilia on the surface of vascular endothelial cells decreased under high wall shear stress and increased under low wall shear stress [41]. It was found that the expression of BMP signaling pathway protein changed with the wall shear stress level. When the blood flow condition of mouse aortic valve changed, the expression of BMP-4 protein was upregulated. At the same time, the adhesion molecules of endothelial cells dependent on BMP-4 protein were also highly expressed, and the downstream Smad1/5/8 protein were phosphorylated [42]. It is worth noting that the pro-inflammatory effect of BMP-4 protein only plays a role when the hemodynamic conditions of the systemic circulation change, and the expression of BMP-4 protein is constant in the pulmonary circulation [43]. Csiszar et al. suggested that in forelimb arteries of aortic banded rats, high intraluminal pressure could promote BMP-2 expression [44]. At the same time, some studies have also shown that in the abnormal hemodynamic environment, there is a cross-linking phenomenon between BMP signaling pathway and other signaling pathways. Under the stimulation of abnormal cerebral blood flow, the number of cilia in vascular endothelial cells decreases, BMP Smad signaling pathway is linked with Wnt/β-catenin signaling pathway, and the expression of β-Catenin closely dependent on BMP Smad signaling pathway expression level, becoming the underlying mechanism leading to atherosclerosis in cerebral vascular wall [41].

## BMP signaling pathway and intracranial aneurysm

IA can be defined as the pathological dilation of cerebral artery. The subarachnoid hemorrhage mostly caused by IA are accompanied by high mortality and disability rate [45]. At present, the etiology of IA has not been clarified thoroughly. From the pathological perspective, IAs are often accompanied by atherosclerotic lesions. Many studies have pointed out that atherosclerotic is an important part of the development of IA [46, 47]. Atherosclerosis is a chronic vascular disease characterized by lipid metabolism disorder, inflammatory response, and calcium deposition. BMP participates in atherosclerosis by regulating endothelial inflammatory response and cell differentiation [48]. BMP-2 and BMP-4 proteins have been found to promote inflammatory response in endothelial cells [49]. In ApoE knockout mice, blocking the BMP signaling pathway can significantly reduce the formation of atherosclerotic plaques; on the contrary, after the activation of BMP signaling pathway, atherosclerotic plaques also increased [50].

Another characteristic pathological change of IA is vascular calcification, which refers to the ectopic deposition of calcium and phosphate in the blood vessels [51]. Gade et al. pointed that calcification is prominently more prevalent in IA, and IA calcification could be classified into two types: nonatherosclerotic type and atherosclerotic type. They also found that in ruptured aneurysms, nonatherosclerotic calcification was more previous. This finding provided us with novel perspective in understanding the role of calcification in IA and searching the new therapeutic targets [52]. Sharma et al. reported that calcification within the aneurysm was strongly associated with aneurysm size, perioperative complications, and the clinical outcome [53]. In the process of vascular calcification, vascular smooth muscle cells, mesenchymal stem cells, and perivascular cells are transformed into osteoblast like cells, and extracellular matrix minerals are gradually deposited in vessel wall. It was found that the expression of BMP increased in the area of vascular calcification, indicating that BMP signaling pathway is involved in the pathological process of vascular calcification [54]. Zhao et al. found that in rat, BMP-2 knockout could significantly block smooth muscle cell calcification [55]. Cheng et al. found that the expression of BMP-2 and bone protein Msx2 of osteoblasts increased in the aorta of diabetic patients. The signal pathway of bmp-2-msx2 may participate in vascular calcification by promoting myofibroblasts to differentiate into osteoblasts [56]. In addition, BMP-2 protein can promote the expression of Runx2 protein, which can promote the calcification of vascular smooth muscle cells through oxidative stress and endoplasmic reticulum stress [57]. When the expression of BMP-2 protein was blocked by matrix glutamate protein, the differentiation of intravascular cells into osteoblasts and chondrocytes decreased [58].

## BMP signaling pathway and cerebrovascular malformation

Cerebrovascular malformation is the congenital development abnormality of cerebrovascular, which is prone to occur in teenagers and middle-aged people, and often results into seriously events such as cerebral hemorrhage and epilepsy.

Cerebral arteriovenous malformations (AVMs) are the most common cerebrovascular malformation, and previous studies have confirmed that BMP signaling pathway was closely related to AVMs. Wang et al. highlighted the specific role of BMP/TGF-β signaling in the etiology of AVMs [59]. Fu et al. performed RNA sequencing analysis on 34 unruptured AVM surgical samples and revealed the abnormal changes in the BMP signaling pathway were significantly associated with microhemorrhage in AVMs, and SMAD6 played an important role in this process. In addition, downregulation of Smad6 expression promoted the formation of endothelial cell tubes with deficient cell-cell junctions and facilitated the acquisition of mesenchymal behavior by endothelial cells [60].

Cerebral cavernous malformation (CCM) is another one of common diseases of cerebrovascular malformation. In recent years, with the progress of imaging technology, the detection rate of CCM has increased a lot, accounting for 10% of all cerebrovascular malformations. Patients with a family history of CCA are often accompanied by gene mutations at chromosome 7q long arm q11 and q22 [61]. Cunha et al. reported that deregulated TGF-β/BMP signaling was strongly related to CCM [62]. Another research reported that the expression of BMP-6 in CCM patients was elevated. In CCM gene knockout mice model, blocking BMP signal pathway can significantly reduce the volume of vascular malformation [63].

Hereditary telangiectasia (HHT), also known as capillary malformation, is a rare genetic disease of cerebrovascular diseases. The incidence rate of HHT is about 1–2 per 100 thousand in population [64]. This kind of cerebral vascular malformation can be seen in all locations of the central nervous system, but most commonly located near the midline of the pons, followed by cerebral cortex and ventricular white matter. At present, the causes of HHT are mostly related to genetic factors, and the common types of gene mutations include ENG gene, ALK1 gene, etc., which can be divided into HHT 1 type and HHT 2 type according to the types of mutant genes [65]. Eng gene and ALK1 gene belong to TGF-β superfamily. It has been reported that the protein mutations of Eng gene include gene deletion, gene insertion, and gene missense mutation, and more than half of ALK1 gene coding proteins belong to gene missense mutation. Among them, ALK1 gene mediates BMP-9 signaling pathway in vascular endothelial cells. Some experiments have applied BMP-9 protein to HHT patients and achieved good results [66]. This therapy is also expected to be further applied in the future clinical work.

## BMP signaling pathway and ischemic cerebrovascular disease

Due to the unique functional characteristics of human brain, brain tissue needs continuous blood flow to supply glucose and oxygen. When the blood flow of intracranial vessels decreases, the brain tissue maintains the steady state of blood flow by self-regulation mechanism. Once this balance is broken, the brain tissue will lead to ischemic event, causing ischemic stroke. With the depletion of oxygen and energy, the brain tissue gradually appears energy metabolism disorder, ion imbalance, and free radical increase and finally leads to irreversible brain damage. Therefore, the key point in the treatment of ischemic cerebrovascular disease lies to the time window. Restoring cerebral blood flow perfusion as early as possible can save the ischemic penumbra in most degree. Once long-term chronic ischemic events occur, promoting angiogenesis in the brain becomes a key factor in the recovery of neurological function. In hypoxia environment, the expression of BMP-4, BMP-7, and BMP-9 protein increased. Drouin et al. suggested that in hypoxic environment, Smad1/5/8 activation was continuous detected for 5 days, so as the BMP-9 mRNA transcription level [67]. Yang et al. found that BMP-2 could increase the motility and migration of smooth muscle cell under hypoxic cultured circumstance [68]. Some studies also showed that the expression of BMP-2 and BMP-4 protein increased in intestinal endothelial cells cultured in vitro under hypoxia and ischemia conditions [69]. Kim et al. found that BMP-9 protein can promote the recovery of neural function in mice with ischemia event [70]. At present, the study of BMP signaling pathway and ischemic cerebrovascular disease is still in the early stage. The exploration of BMP signaling pathway is conducive to clarify the mechanism of ischemic cerebrovascular events.

## Conclusions

Different BMPs together with various corresponding downstream cascade molecules determine the complexity of BMP signal pathway. BMP signal pathway plays a wide role in different tissues and cell environments. In addition to the BMP signal pathway, BMP signaling pathway is also cross-linked with other signaling pathways to exert a role in human body. In this review, we have summarized the biology and mechanism of BMP signaling pathway played in cerebrovascular system. A large number of human and animal experiments show that TGF-β superfamily and BMP are closely related to cerebrovascular system. This review has revealed that BMP signal pathway broadly participated in cerebrovascular neovascularization and typical CVDs, such as IA, CCM, and ischemic cerebrovascular event.

However, the current knowledge regarding the BMP signal pathway in different tissues has been mostly derived from animal models while much less clinical investigations were implemented. At the same time, we have also noticed that the researches focusing on the relationship between BMP signal pathway and cerebrovascular diseases are still limited. For some certain BMP s and the following cascade molecules, the exact roles they played in cerebrovascular system are still controversial, and the current situation requires more detailed studies targeting this topic. The in-depth study of BMP signaling pathway provides us with a new perspective to explore the pathogenesis and development mechanism of cerebrovascular diseases and also is expected to bring new perspective to the diagnosis and treatment of cerebrovascular diseases.

## Data Availability

The datasets used and analyzed during the current study are available from the corresponding author on reasonable request.

## Abbreviations

IA: Intracranial aneurysm
CVD: Cerebrovascular diseases
BMP: Bone morphogenetic protein
TGF-β: Transcription growth factor superfamily β
GDF: Growth and differentiation factor
BMPRI: BMP receptor I
BMPRII: BMP receptor II
ACVR2A: Activin receptor 2A
ACVR2B: Activin receptor 2B
R-Smad: Regulated Smad
I-Smad: Inhibitory Smad
MAPK: Mitogen activated protein kinase
PKC: Protein kinase C
PI3K: Phosphoinositide 3 kinase
CCM: Cerebral cavernous malformation
HHT: Hereditary telangiectasia
